# Genome sequencing of *Elaeocarpus* spp. stem blight pathogen *Pseudocryphonectria elaeocarpicola* reveals potential adaptations to colonize woody bark

**DOI:** 10.1186/s12864-024-10615-5

**Published:** 2024-07-24

**Authors:** Yuchen Yang, Dianguang Xiong, Danyang Zhao, Huayi Huang, Chengming Tian

**Affiliations:** 1 State Key laboratory of Efficient Production of Forest Resources, Beijing Forestry University, Beijing, 100083 China; 2 Guangdong Provincial Key Laboratory of Silviculture, Protection and Utilization, Guangdong Academy of Forestry, Guangzhou, 510520 Guangdong China

**Keywords:** Fungal genomic, *Pseudocryphonectria elaeocarpicola*, Pathogenic factors, CAZymes, Secondary metabolism, Stem blight

## Abstract

**Background:**

*Elaeocarpus* spp. stem blight, caused by *Pseudocryphonectria elaeocarpicola*, is a destructive disease, which will significantly reduce the productivity and longevity of *Elaeocarpus* spp. plants, especially in the Guangdong Province of China. However, few information is available for *P. elaeocarpicola*. To unravel the potential adaptation mechanism of stem adaptation, the whole genome of *P. elaeocarpicola* was sequenced by using the DNBSEQ and PacBio platforms.

**Results:**

*P. elaeocarpicola* harbors 44.49 Mb genome with 10,894 predicted coding genes. Genome analysis revealed that the *P. elaeocarpicola* genome encodes a plethora of pathogenicity-related genes. Analysis of carbohydrate-active enzymes (CAZymes) revealed a rich variety of enzymes participated in plant cell wall degradation, which could effectively degrade cellulose, hemicellulose and xyloglucans in the plant cell wall and promote the invasion of the host plant. There are 213 CAZyme families found in *P. elaeocarpicola*, among which glycoside hydrolase (GH) family has the largest number, far exceeding other tested fungi by 53%. Besides, *P. elaeocarpicola* has twice as many genes encoding chitin and cellulose degradation as *Cryphonectria parasitica*, which belong to the same family. The predicted typical secreted proteins of *P. elaeocarpicola* are numerous and functional, including many known virulence effector factors, indicating that *P. elaeocarpicola* has great potential to secrete virulence effectors to promote pathogenicity on host plants. AntiSMASH revealed that the genome encoded 61 secondary metabolic gene clusters including 86 secondary metabolic core genes which was much higher than *C. parasitica* (49). Among them, two gene cluster of *P. elaeocarpicola*, cluster12 and cluster52 showed 100% similarity with the mycotoxins synthesis clusters from *Aspergillus steynii* and *Alternaria alternata*, respectively. In addition, we annotated cytochrome P450 related enzymes, transporters, and transcription factors in *P. elaeocarpicola*, which are important virulence determinants of pathogenic fungi.

**Conclusions:**

Taken together, our study represents the first genome assembly for *P. elaeocarpicola* and reveals the key virulence factors in the pathogenic process of *P. elaeocarpicola*, which will promote our understanding of its pathogenic mechanism. The acquired knowledge lays a foundation for further exploration of molecular interactions with the host and provide target for management strategies in future research.

**Supplementary Information:**

The online version contains supplementary material available at 10.1186/s12864-024-10615-5.

## Background

Blight and canker diseases have caused significant adverse effects on the production and management of woody plants worldwide. Among numerous pathogenic fungi, Cryptomeriaceae, a pathogenic family within Diaporthales, contains a variety of pathogenic fungi that cause blight and canker diseases [1, 2]. The most notorious species is *Cryphonectria parasitica* which causes chestnut blight throughout the world, reducing the longevity and productivity of infected trees [3]. *Chrysoporthe cubensis*, *Chrysoporthe deuterocubensis*, and *Chrysoporthe austroafricana* are the causal agent of eucalypt (*Eucalyptus* spp.) canker diseases in South America, Asia and Africa, respectively [4]. Recently, *Pseudocryphonectria elaeocarpicola* is found to cause severe stem blight on *Elaeocarpus* spp. in China [5].

**Fig. 1:**
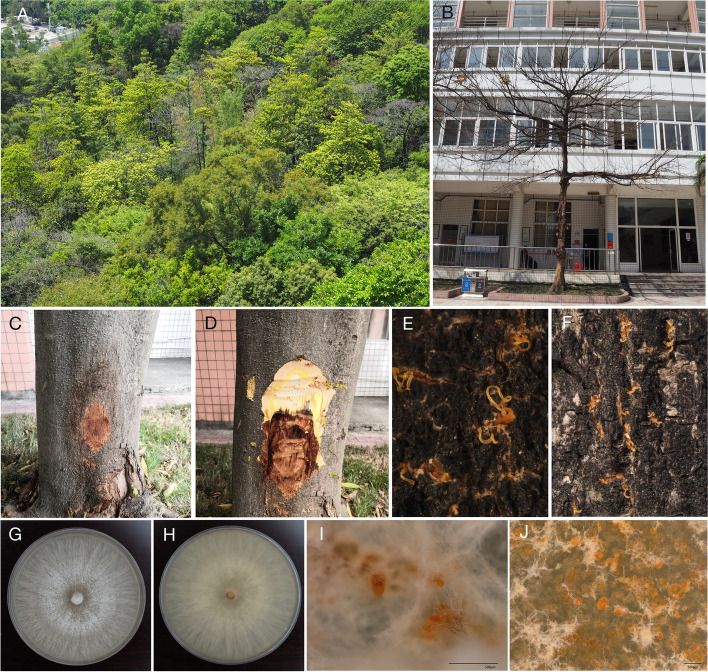
Symptoms caused by *Pseudocryphonectria elaeocarpicola* on *Elaeocarpus* trees. **A**, **B** Dead trees.; **C**, **D** Symptom of * P. elaeocarpicola* on * Elaeocarpus* bark.; **E**, **F** Orange conidial tendrils formed on the cankered barks.; **G**-**J** Morphology of *P. elaeocarpicola* from PDA

*Elaeocarpus* spp. is an important garden tree species with significant economic and ecological value in Guangdong Province, China. However, *Elaeocarpus* spp. stem blight disease caused by *P. elaeocarpicola* is a restraint to the growth of *Elaeocarpus* spp., which threaten the landscape greening in Guangdong Province seriously (Fig. 1A, B). The disease mainly develops on areas of the trunk or branches of the host (Fig. 1C). The bark of infected branches appears scorched and withered (Fig. 1D). Besides, *P. elaeocarpicola* forms orange conidial tendrils on the host bark at the late stages of the disease cycle (Fig. 1E-J). Moreover, *P. elaeocarpicola* significantly shorten the lifespan and productivity of the infected trees, and most of affected trees died within five months [5]. However, the disease cycle, epidemiology, histocytology and molecular mechanism underlying the pathogenicity of *Elaeocarpus* spp. stem blight disease are scarcely reported. Therefore, predicting the virulence factors of *P. elaeocarpicola* through whole genome sequencing is necessary to study the pathogenic mechanism and develop the more effective disease management strategies.

The rapidly developing new generation sequencing technology provides high-quality, low-cost genomic data that can be used for diverse analyses. To date, genomic research has been performed on hundreds of multiple plant pathogenic fungi [6–8]. Genomic research has greatly contributed to the prediction of pathogenic fungal virulence factors and the analysis of invasion mechanism [9]. In addition, many bioinformatics computational tools and well-established fungal databases provide greater assistance in mining virulence factors in pathogenic fungal genome sequences. The commonly used analysis tools and databases are as follows: CAZydatabase, antiSMASH, Cytochrome P450 Engineering Database, SignalP, EffectorP and Transporter Classification Database [10–14]. With the help of these tools and databases, researchers are able to study the interactions between pathogenic fungi and hosts, thus providing more precise research objectives for the study of pathogenic fungal pathogenesis and control management.

The disease establishment of pathogenic fungi on host plants depends on diverse virulence factors, including cell wall degrading enzymes, effector proteins, secondary metabolites, transporter proteins and so on [15]. Pathogenic fungi require complete and complex gene regulatory networks to regulate these virulence factors to accomplish host penetration, nutrient acquisition, prevention or inhibition of host defense, and even kill plant tissue. Therefore, virulence factors are essential for the interaction between fungal pathogens and host. The carbohydrate-active enzymes (CAZymes) are crucial factors, which secreted by plant pathogenic fungi in the initial infection process [16–18]. CAZymes can break down the components of plant cell wall (cellulose, hemicellulose, xylan, xyloglucan, mannan and pectin), which enable pathogens to colonize host plants [19]. Therefore, these enzymes are particularly important during the early stages of infection for plant pathogenic fungi as well as the late infection stages [20]. Although the enzymatic activity is critical for the role of CAZymes, studies of *Botrytis cinerea*, *Sclerotinia sclerotiorum*, and *Mycosphaerella graminicola* have shown that CAZymes have a positive relationship with virulence and act as virulence factors [21–24]. After colonization on host plants, pathogens attack plant cells through a variety of strategies including secondary metabolites (SMs), cytochrome P450 monooxygenases and transporters [25–28].

Fungal pathogens produce a range of SMs with strong biological activities during the secondary metabolism, which play important roles in the development and pathogenesis. The genes encoding SMs are usually clustered together [29], and this chromosomal architecture has facilitated the prediction and mining of secondary metabolism gene clusters in fungal genomes. Secondary metabolism gene clusters are minimally composed of backbone genes and tailoring enzymes genes. Backbone genes are the most central and critical genes in gene clusters, mainly including non-ribosomal peptide synthetase (NRPS) genes, terpene synthase genes and polyketide synthase (PKS) genes. Tailoring enzymes mainly include methyltransferases, cytochrome P450 monooxygenases, hydroxylases and epimerases [29]. Mycotoxins synthesized under the encode of secondary metabolism gene clusters and core genes have been proved to play key roles in pathogenic processes and can directly damage or kill host plant tissues. The well-known mycotoxin include T-toxin, deoxynivalenol (DON), ochratoxin A (OTA), aflatoxin and so on [30–33]. In addition to attacking host plants directly, secondary metabolites can also inhibit host immune defense mechanisms. Cytochrome P450 related enzyme (CYPs or P450) are also involved in the synthesis of secondary metabolites [34, 35]. CYPs are proteins containing heme and one of the largest protein families, present in all biological kingdoms [36]. Moreover, fungal CYPs also participate in multiple biological processes, including primary metabolism, promoting fungal adaptations to various ecological niches and denitrification. Notably, CYPs play key catalytic abilities in the biosynthesis of secondary metabolites. The reactions catalyzed by CYPs greatly enriches the chemical structure and biological activity of secondary metabolites [36].

Besides, transport proteins are a large group of membrane proteins that mediate the exchange of chemicals as well as signals inside and outside biological membranes. Meanwhile, active efflux by major facilitator superfamily (MFS) transporters or ATP-binding cassette (ABC) can develop resistance to various toxic compounds such as SMs, antibiotics and fungicides [37–39]. Furthermore, in order to survive in a wide variety of environments, fungi often use extracellular proteins to decompose nutrients, obtain metal ions, and mediate interactions with the host [40]. Notably, pathogens have the ability to secrete enzymes and other proteins to overcome the host defense responses or directly kill the host [41, 42]. Further correspondence studies based on the predicted results of these virulence factors could study the pathogenic mechanism of novel plant pathogens more effectively and provide ideas and directions for further development of prevention and control strategies.

In this study, we sequenced the genome of the *P. elaeocarpicola*, which caused *Elaeocarpus* spp. stem blight. Comparative analyses showed that the *P. elaeocarpicola* genome encodes a variety of functional genes, including a lot of potential virulence factors. These virulence factors most likely play important functions in the invasion and colonization of host plants by *P. elaeocarpicola*. Notably, gene families related to cellulase, hemicellulase, and secondary metabolic are expanded in *P. elaeocarpicola*. The analysis of CAZymes shows that *P. elaeocarpicola* has a strong potential to secrete cell wall degrading enzymes. Meanwhile, the secondary metabolic genes in *P. elaeocarpicola* have great potential to play a dominant role in the biological processes of development and virulence. In addition, a lot of toxic effector proteins, CYPs and transcription factors with known homologous pathogenic effects are annotated in *P. elaeocarpicola* genome. In summary, the analysis results of *P. elaeocarpicola* genome reported in this article provide valuable information for studying the pathogenesis of this novel blight pathogen.

## Results

### Genome characteristics of *P. elaeocarpicola*

**Fig. 2:**
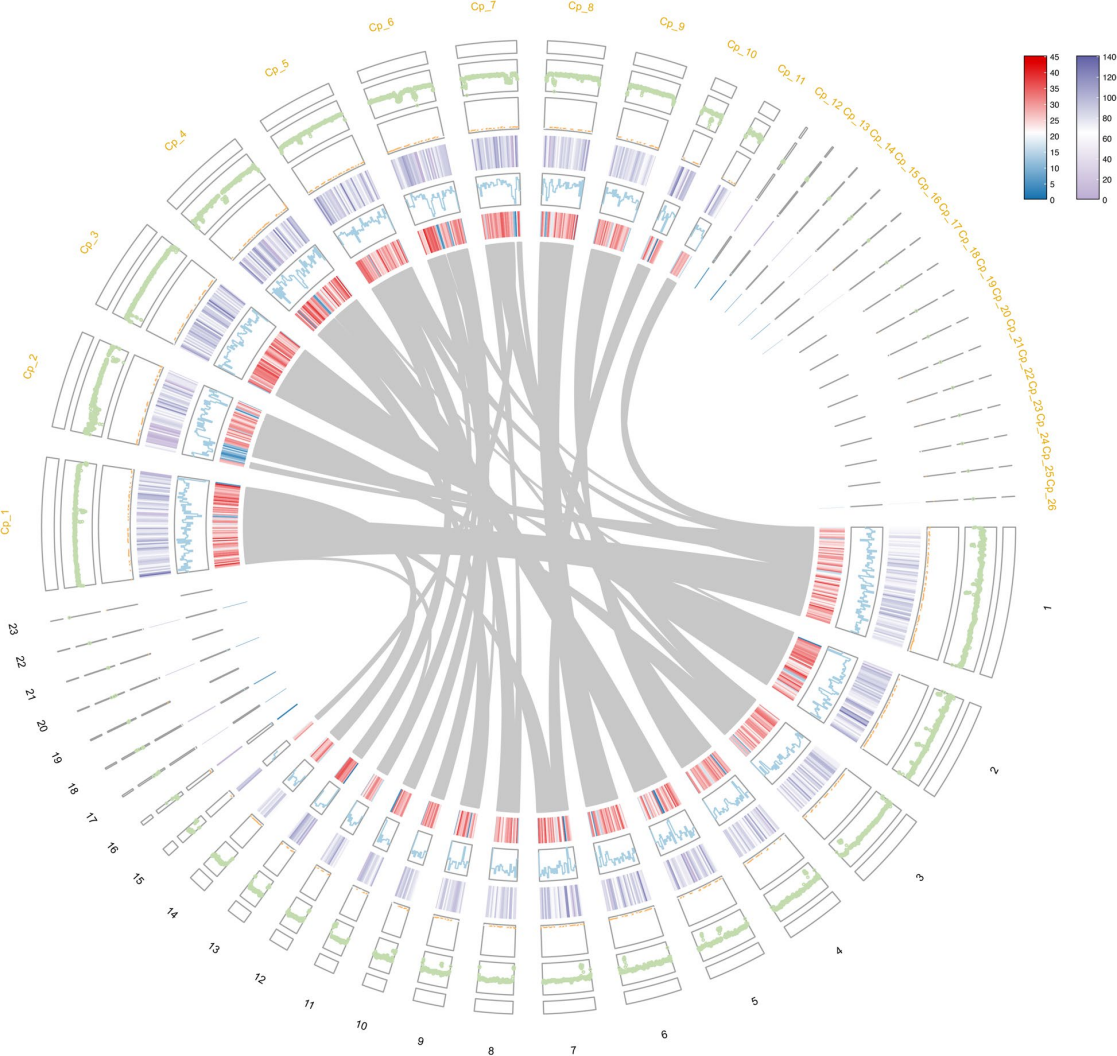
Circos-plot of genome features of *P. elaeocarpicola. *The circles from outside going in represent genomic synteny and comparison between *P. elaeocarpicola* and *Cryphonectria parasitica*, gene density, CDS density, GC skew, GC ratio, 1–23 represent *P. elaeocarpicola* chromosome skeleton, Cp_1-Cp_26 represent *C. parasitica* chromosome skeleton

 Genome of *P. ela*e*ocarpicola*, a blight pathogen isolated from *Elaeocarpus* spp., was sequenced de novo and assembled to a genome size of 44.49 Mb. The genome sequence contained 23 scaffolds and coverage of 98.2% (Table 1; Fig. 2). The length of N50 was 3.42 Mb, and the largest scaffold was 8.5 Mb, the smallest scaffold was 4291 bp. The GC content was 53.18%, and length of the repetitive sequences in *P. elaeocarpicola* genome was 3.66 Mb, representing 8.72% of the genome (Table 1; Fig. 2). In addition, a total of 10,894 protein-coding genes were predicted in the *P. elaeocarpicola* genome. The average gene length of the *P. elaeocarpicola* genome is 1775 bp, with an intron length of 145 bp, which is similar to other Ascomycota species [28, 43]. The *P. elaeocarpicola* genome size is comparable to other filamentous fungi such as *Valsa mali* (44.7 Mb), *C. parasitica* (43.9 Mb) and *Magnaporthe oryzae* (42.7 Mb).

**Table 1:** Genome features of the *Pseudocryphonectria elaeocarpicola*

Feature	*P. elaeocarpicola*
Number of scaffolds	23
Coverage (%)	98.2
Length of genome assembly (Mbp)	44.49
Genome depth	28.78
N50(Mbp)	3.42
GC content (%)	53.18
Repeat size (Mbp)	3.66
Repeat rate (%)	8.7155
Gene number	10894
Average gene length (bp)	1775.16
Average exon size (bp)	516.4
Average coding sequence size (bp)	1499.61
Average intron size (bp)	144.72
ncRNA	1242

**Fig. 3:**
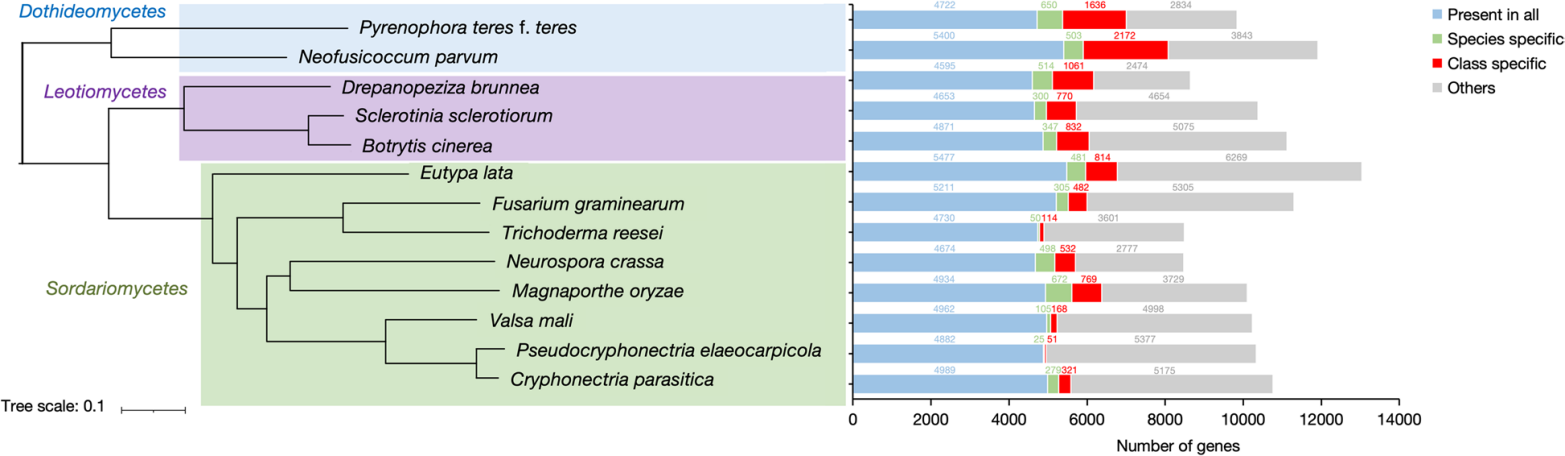
Phylogenetic relationship and orthologous gene cluster. A maximum likelihood phylogenetic tree was constructed based on 2925 single-copy ortholog genes showing the phylogenetic relationship of *P. elaeocarpicola* and 12 reference fungi. The colored bars and numbers represent orthologous proteins identified using Orthofinder for each fungal genome

Furthermore, to explore the evolutionary relationship and gene family of *P. elaeocarpicola*, we clustered the proteomes of *P. elaeocarpicola* with those of 12 typical stem and foliar fungi with different lifestyles using Orthofinder, and constructed a phylogenetic tree based on the results of gene family analysis (Fig. 3). The results of the ortholog family analysis showed that a total of 2925 single-copy orthologs were conserved in all analyzed fungi. There are 4882 genes that can be clustered into gene families in *P. elaeocarpicola*, and the number of gene families is 51. In addition, a total of 25 species-specific proteins were identified in *P. elaeocarpicola*, distributed among 10 unique gene families. Besides, the resulting phylogenomic tree shows that *P. elaeocarpicola* is closely related to *C. parasitica*, which matches the previous reports [5]. In addition, we performed synteny analysis of *P. elaeocarpicola* with *C. parasitica.* Synteny analysis revealed higher sequence similar between *P. elaeocarpicola* and *C. parasitica* (Fig. 2).

### Genome functional annotation of *P. elaeocarpicola*

**Fig. 4:**
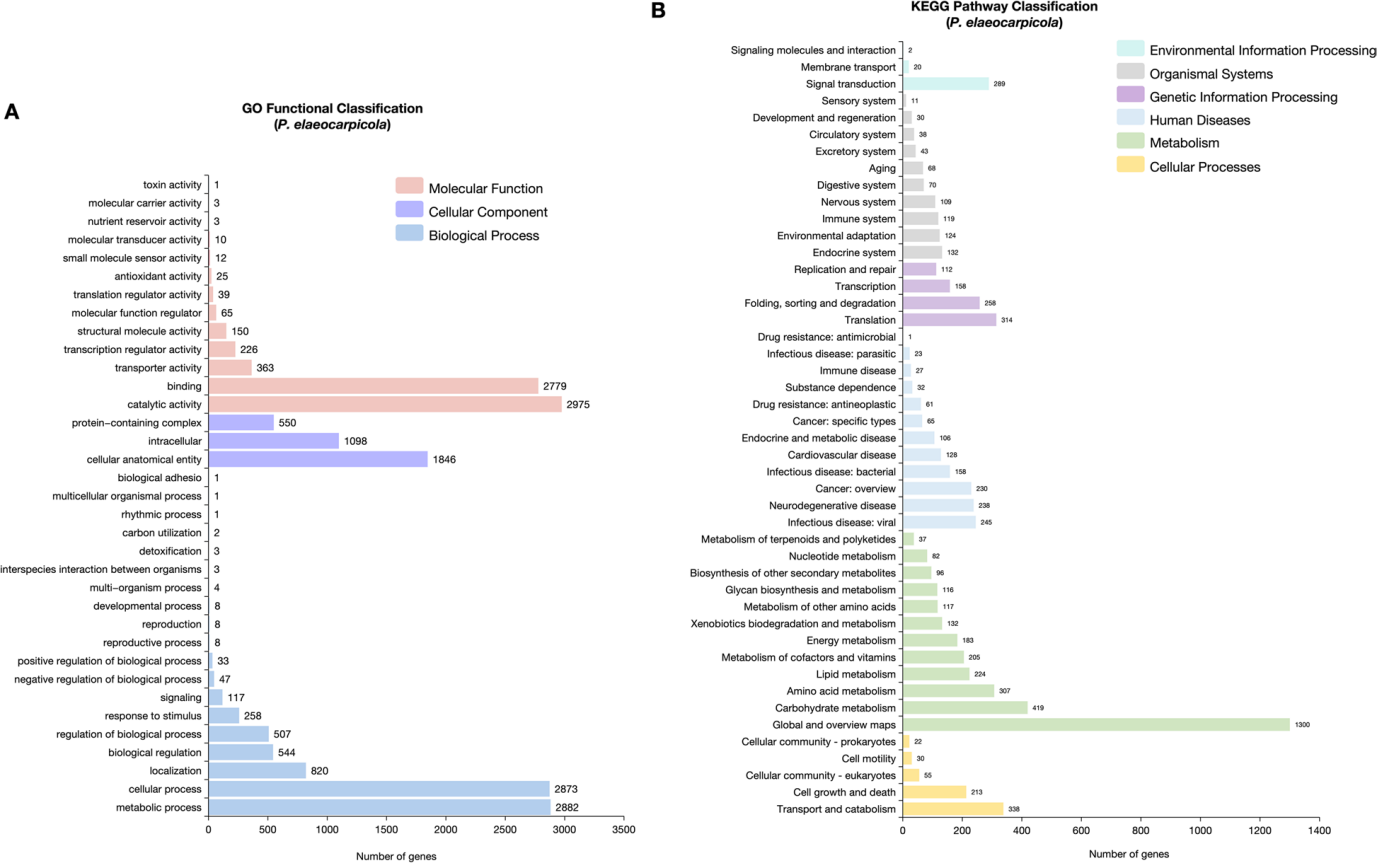
Analysis of genome annotation and metabolic pathway of *P. elaeocarpicola.* **A** Gene Ontology (GO) annotation of *P. elaeocarpicola* genome.; **B** KEGG pathway annotation of *P. elaeocarpicola* genome

To further explore the function of the *P. elaeocarpicola*, Gene Ontology (GO) and Kyoto Encyclopedia of Genes and Genomes (KEGG) database were used to predict the annotations of genes in *P. elaeocarpicola* (Fig. 4).

A total of 6011 genes were annotated in the GO database, which can be categorized into three subcategories based on the function of each gene: biological processes (19 branches), cellular components (3 branches), and molecular functions (13 branches; Fig. 4A). In the biological process subcategories, the three largest branches are metabolic, metabolic process (2882), cellular process (2873), and localization (820) are the three largest branches. Among the cellular component subcategories, the proportion of cellular anatomical entities (1846) is the highest. In the molecular function subcategories, catalytic activity (2975), binding (2779), and transporter activity (363) are ranked in the top three, respectively.

The results of KEGG pathway mapping shows that a total of 4463 genes were correspond to 46 metabolic pathways (Fig. 4B). The main pathways, in descending order of the number of genes involved, are as follows: MAPK signaling pathway – yeast (ko04011; 65 genes; Environmental Information Processing; Signal transduction), mTOR signaling pathway (ko04150; 44 genes; Environmental Information Processing; Signal transduction); ribosome (ko03010; 111 genes; Genetic Information Processing; Translation), spliceosome (ko03040; 96 genes; Genetic Information Processing; Translation); amyotrophic lateral sclerosis (ko05014; 158 genes; Human Diseases; Neurodegenerative disease), Pathways of neurodegeneration - multiple diseases (ko05022; 155 genes; Human Diseases; Neurodegenerative disease); metabolic pathways (ko01100; 1260 genes; Metabolism; Global and overview maps ), biosynthesis of secondary metabolites (ko01100; 465 genes; Metabolism; Global and overview maps ); cell cycle - yeast (ko04111; 89 genes; Cellular Processes; Cell growth and death), meiosis-yeast (ko04113; 79 genes; Cellular Processes; Cell growth and death).

### *P. elaeocarpicola* genome has advantages in cellulose degradation

**Fig. 5:**
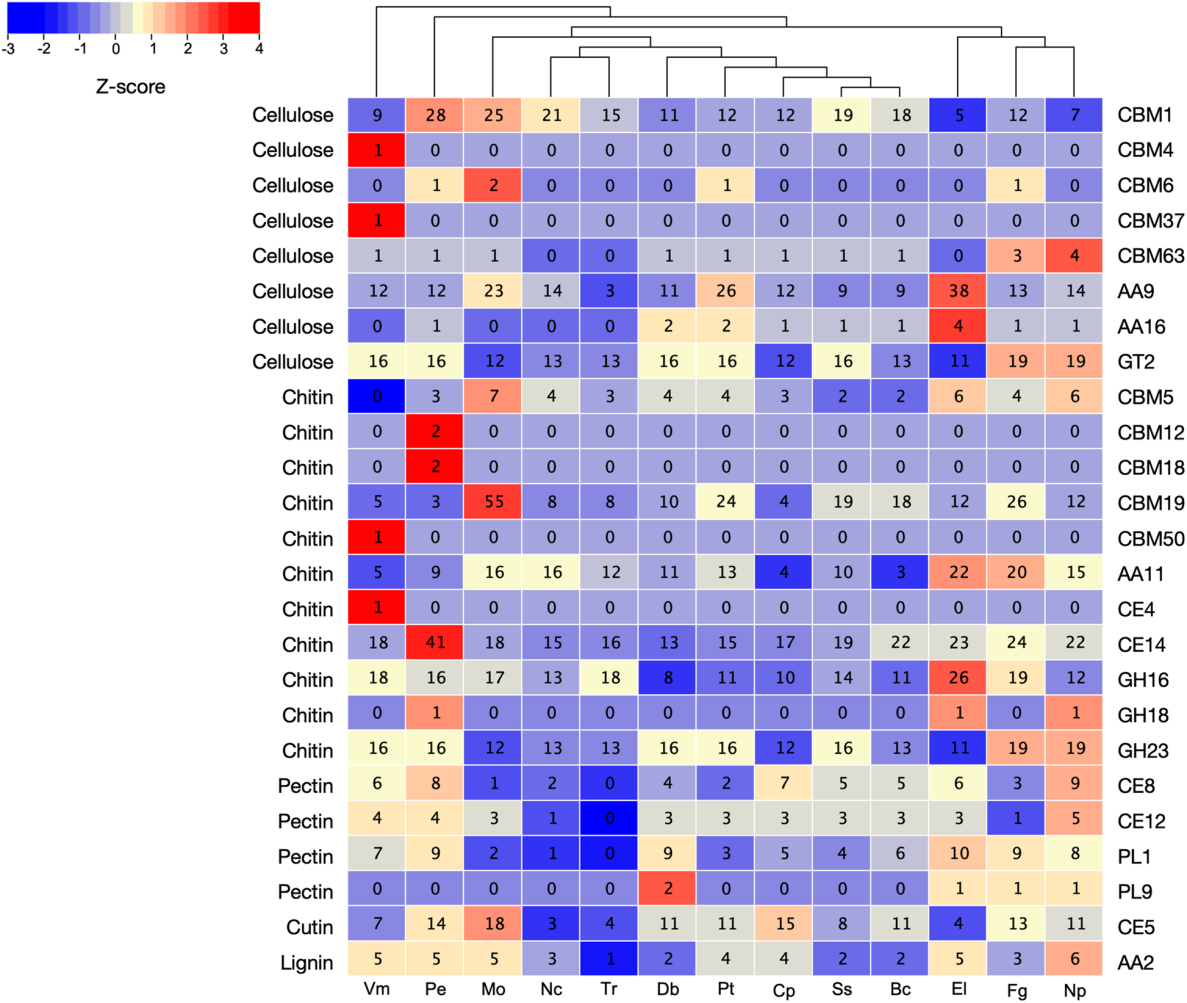
Hierarchical clustering of CAZyme families from *P. elaeocarpicola* and 12 other fungal genomes. Right side of heatmap showing the characterized subfamilies of CAZymes. GH, glycoside hydrolase; GT, glycosyl transferase; CE, carbohydrate esterase; AA, auxiliary activities; CBM, carbohydrate-binding module; PL, polysaccharide lyase. Fungal names (left to right): Vm, * Valsa mali*; Pe, * Pseudocryphonectria elaeocarpicola* ; Mo, * Magnaporthe oryzae* ; Nc, * Neurospora crassa* ; Tr, * Trichoderma reesei* ; Db, * Drepanopeziza brunnea* ; Pt, * Pyrenophora teres* f. * teres* ; Cp, *Cryphonectria parasitica* ; Ss, * Sclerotinia sclerotiorum* ; Bc, * Botrytis cinerea* ; El, * Eutypa lata*; Fg, * Fusarium graminearum* ; Np, * Neofusicoccum parvum*. Overrepresented (yellow to red) and underrepresented (yellow to blue) domains are depicted as Z-scores for each family. Data are from this study and from the carbohydrate-active enzyme database (http://www.cazy.org/)

The plant cell wall consists of structural proteins, aromatic compounds and carbohydrates such as pectin, cellulose, and hemicellulose, which acts as a front line against pathogenic fungi [44, 45]. CAZymes secreted by pathogenic fungi play a crucial role in plant cell wall degradation and are therefore important virulence factors [16]. CAZymes are composed of proteins with the ability to modify, degrade, or generate glycosidic bonds and are classified into six main classes: glycoside hydrolases (GHs), glycosyltransferases (GTs), polysaccharide lyases (PLs), carbohydrate esterases (CEs), carbohydrate-binding modules (CBMs), and auxiliary activities (AAs) [11, 46]. Based on the importance of CAZymes, we predicted the CAZymes content in *P. elaeocarpicola* genome and compared it with those of 12 other representative fungi (Fig. 5, Table S1).

The CAZymes identification the existence of 213 different CAZyme families. Among them, the GH family (113 GH families) is the most abundant, followed by the GT family (41 GT families). In the GH family, the *P. elaeocarpicola* genome contained the highest number of GH5, GH43, and GH16 families, which were much higher than other tested fungi (Table S1). GH16 family is a large and taxonomically diverse family of glycosidases and transglycosidases that participate in various physiological processes, including in the degradation or remodeling of cell wall polysaccharides, nutrient absorption, invasion and pathogenesis, and regulation of immune responses. The subfamilies of GH16 in *P. elaeocarpicola* genome include GH16-1, GH16-3, GH16-4, GH16-10, GH16-18, GH16-19, GH16-20, and GH16-23, of which the genes coding for the subfamilies of GH16-1, GH16-18, and GH16-20 ranked the top three in terms of number. GH16-20 is a functional subfamily composed of plant enzymes specific for xyloglucans [47]. The enzymes of this subfamily mainly consist of two main groups: xyloglucan endo-transglycosylases and xylan endohydrolases [48]. Xyloglucans are widely distributed in vascular plants and are the main component of the primary wall of all dicots and some monocots [48]. As shown in Fig. 5, P. *elaeocarpicola* contains large number of genes encoding cellulose degradation, with a total of 59 genes. Besides, GH5-7, which is important for hemicellulose degradation, was also identified (Table S1). These results indicate that *P. elaeocarpicola* is able to degrade cellulose, hemicellulose, and xyloglucans in host plant cell walls effectively.

Hierarchical clustering analysis showed that *P. elaeocarpicola* has unique CAZymes profile (Fig. 5). Remarkably, *P. elaeocarpicola* genome contain the largest number of CBM1, CBM12, CBM18, and CE14 families among all tested fungi. CBM1 family is widely present in fungal cellulases and can promote cellulose degradation by increasing the local concentration of cellulase [49, 50]. The number of cutinases genes (CE5 family) in *P. elaeocarpicola* was similar to *C. parasitica*, which is consistent with the fact that pathogenic fungi of Cryptomeriaceae family penetrate wounds by degrading plant cuticles. In addition, compared with *C. parasitica*, *P. elaeocarpicola* contains comparable number of genes involved in pectin, cuticle and lignin degradation, but the number of genes encoding degradation of chitin and cellulose were much more. These results showed that *P. elaeocarpicola* may possess a greater potential to degrade chitin and cellulose in comparison with *C. parasitica.*

### *P. elaeocarpicola* genome is rich in potentially virulent related secret proteins

 It has been shown that fungi accomplish adaptation to the host environment, acquisition of nutrients and defense against immune responses through secreted proteins and other molecules. Especially, the secreted proteins produced by pathogenic fungi can facilitate colonization of host tissue and even kill host cells [40]. Therefore, the identification and functional annotation of pathogenic fungi secretome is important for identifying potential virulence factors. In this study, SIGNALP and TMHMM were used to predict secreted proteins in the genome of *P. elaeocarpicola*. There are 10,894 predicted genes encoding proteins in the *P. elaeocarpicola* genome with 841 putative secreted proteins, accounting for 7.72% of the genome. Functional enrichment showed that 367 secreted proteins could be assigned GO terms. Among them, proteins involved in hydrolase activity, hydrolyzing O-glycosyl compounds, pectin metabolic process, carbohydrate catabolic process, and cell wall polysaccharide metabolic process are significantly overrepresented in the *P. elaeocarpicola* secretome (Fig. S1). In addition, functional analysis showed that, *P. elaeocarpicola* secretome contains homologs of known pathogenic effectors of other plant pathogens (Table 2), including Gel1 and *Mo*PMO9A of *M. oryzae* [51, 52], Fgl1 and FgKP4L-2 of *Fusarium graminearum* [53, 54], and the pelB of *Colletotrichum gloeosporioides* and so on [55–58].

**Table 2:** Putative effectors showing homology to known fungal effectors

*P. elaeocarpicola*	Annotation	Domain	Reference
GME4321_g	family 72 glycoside hydrolase	Glucanosyltransferase (IPR004886)	Samalova, Marketa et al. (2017) [52]
GME4462_g	DnaJ-domain-containing protein	Chaperone DnaJ (IPR012724)	Wang, Jie et al. (2016) [57]
GME10643_g	glycosylhydrolase family 61-1	Auxiliary Activity family 9	Martinez-D'Alto, Alejandra et al. (2023) [51]
GME9214_g	family 5 carbohydrate esterase	Cutinase/acetylxylan esterase (IPR000675)	Gong, Yingdi et a (2022) [55]
GME5618_g	alpha/beta-hydrolase	Alpha/Beta hydrolase fold (IPR029058)	Sella, Luca et al. (2014) [54]
GME5964_g	family 1 polysaccharide lyase	Pectin lyase family (IPR045032)	Yakoby, N et al. (2001) [58]
GME4212_g	killer toxin	Killer toxin Kp4/SMK (IPR011329)	Lu, Shunwen, and Justin D Faris (2019) [53]
GME4353_g	family 5 carbohydrate esterase	Cutinase/acetylxylan esterase (IPR000675)	Lee, Miin-Huey et al (2010) [56]

Based on secreted proteins, a total of 236 effector proteins in the *P. elaeocarpicola* genome were predicted by EffectorP. Of these 236 effector proteins, 169 proteins were annotated as apoplastic effector, 59 proteins were annotated as cytoplasmic effector, and eight proteins were annotated as apoplastic or cytoplasmic effector. Subsequently, we matched 236 effector proteins to the Non-Redundant Protein Database (NR), and 209 proteins were matched. Among them, 171 proteins were annotated to *C. parasitica*, of which 63 proteins were functionally known with the identity majority above 81.8% (Table S2). This result is consistent with the phylogenetic analysis of *P. elaeocarpicola.* Among these 171 effector proteins, a total of 34 proteins were annotated as GHs, encompassing 10 families, with GH28 (12 proteins) accounting for the largest proportion. GH28 family contains polygalacturonases (PG), rhamnogalacturonases (RG), and xylogalacturonases (XG), which have the function of degrading the pectin layer of plant cell walls. Many GH28 family members have been identified and characterized as virulence factors for pathogenic fungi. In addition, we have annotated four GH7 family proteins in the *P. elaeocarpicola* genome (Table S2).

Additionally, 27 effector proteins were specific in *P. elaeocarpicola.* Function prediction was performed for 27 proteins using the InterProScan tool, among which GME8245_g (Tripeptidyl-peptidase I and related peptidases, IPR050819), GME97_g (Cerato-ulmin hydrophobin family, IPR010636) and GME6290_g (HeLo domain superfamily, IPR038305) were functionally annotated. Cerato-ulmin hydrophobin family may mediate contact and communication between fungi and environment [59]. In addition, this family includes cryparin, a cell-surface-associated hydrophobin secreted by filamentous fungi, trihydrophilin in the ergot *Claviceps fusiformis*, and cerato-ulmin, secretory toxin hydrophilin associated with Dutch elm disease [60–62]. These specific proteins most likely determine the specific function of *P. elaeocarpicola* and have high research value for further research on the evolution of *P. elaeocarpicola*. Taken together, these results suggest that *P. elaeocarpic*o*la* would promote pathogenicity to host plants by secreting a large abundance of putative effector proteins.

### The secondary metabolism genes of* P. elaeocarpicola* are particularly abundant

**Fig. 6:**
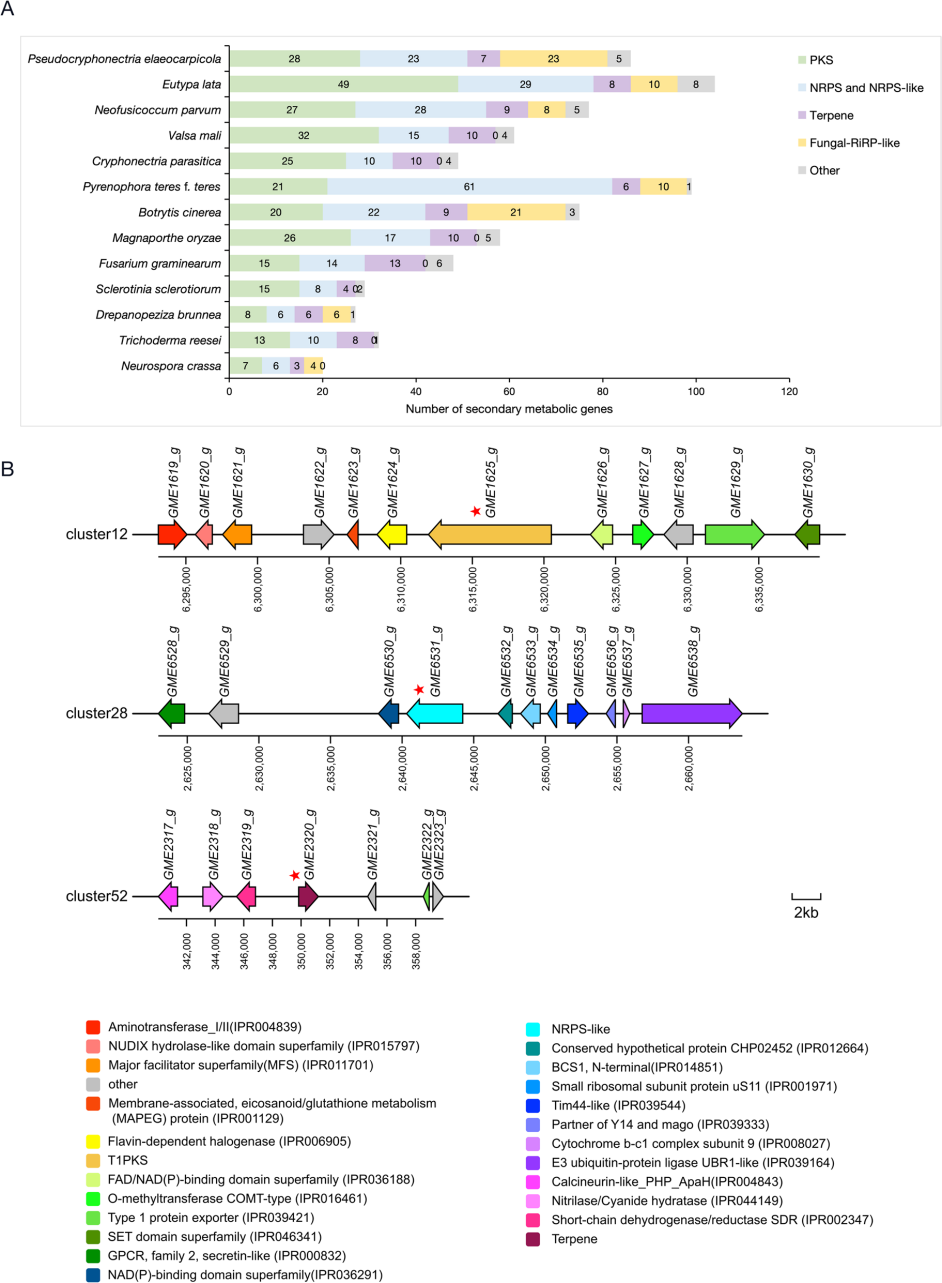
The core secondary metabolism genes and pathogenic related homologous gene clusters in *P. elaeocarpicola. ***A **The core genes involved in the biosynthesis of secondary metabolites form *P. elaeocarpicola* and 12 other fungal genomes. NRPS and NRPS-like (Non-Ribosomal Peptide Synthase and NRPS-like fragment), PKS (Type I Polyketide Syntha and Type III Polyketide Synthase), Terpene, Fungal-RiPP-like (Fungal RiPP-likes), Other (indole, orf2_PTase, phosphonate-like, phosphonate). Data are from this study and from the antiSMASH fungal v7.0 (https://fungismash.secondarymetabolites.org/#!/start); **B** The line with the arrow indicates the scheme of the cluster12 (GME1619_g ~ GME1630_g), cluster28 (GME6528_g ~ GME6538_g), and cluster52 (GME2317_g ~ GME2323_g) of *P. elaeocarpicola*. Domains were predicted using the InterProScan tool. Different color boxes indicate different domain categories. The red star represents the core gene. Bar = 2 kb

 The secondary metabolites (SMs) secreted by fungi play a crucial role in nutrient acquisition, chemical warfare, communication, and ecological interactions. In phytopathogens, phytotoxic SMs are the key weapons during the invading of host plant, mainly including polyketide synthases (PKSs), non-ribosomal peptide synthetases (NRPSs) and terpenes. AntiSMASH7.1 was applied to identify genes involved in secondary metabolite biosynthesis in the genome of *P. elaeocarpicola* (Table 3 and Table S3). The identification results showed that *P. elaeocarpicola* encodes a wide variety of secondary metabolism core enzymes, including 28 PKSs, 23 NRPSs and putative NRPS-like enzymes, seven terpenes, 23 Fungal-RiRP-likes, and five other enzymes. These core enzymes are distributed in 61 secondary metabolism gene clusters. A total of 44 transporter genes were identified in the 61 secondary metabolism gene clusters. As shown in Fig. 6A, we further compared the SMs genes of *P. elaeocarpicola* to 12 typical stem and foliar fungi with different lifestyles. Notably, *P. elaeocarpicola* genome contains abundant SMs genes among all tested fungi. These results indicated that *P. elaeocarpicola* have the great potential for SMs production.

**Table 3:** The core genes involved in the biosynthesis of secondary metabolites

Species	Scaffold	PKS	NRPS and NRPS-like	Terpene	Fungal-RiRP-like	Other	Total
*P. elaeocarpicola*	Scaffold1	9	3	2	7	0	
	Scaffold2	6	3	1	3	3	
	Scaffold3	0	1	1	3	0	
	Scaffold4	3	3	1	0	2	
	Scaffold6	3	2	0	5	0	
	Scaffold7	1	1	0	1	0	
	Scaffold8	0	1	1	0	0	
	Scaffold9	3	4	0	1	0	
	Scaffold10	1	0	1	1	0	
	Scaffold11	1	2	0	1	0	
	Scaffold12	1	2	0	0	0	
	Scaffold13	0	0	0	1	0	
	Scaffold14	0	1	0	0	0	
	Total	28	23	7	23	5	86

The homology analysis of *P. elaeocarpicola* secondary metabolism gene clusters showed that out of 61 secondary metabolism gene clusters, a total of seven (more than 50% similarity) gene clusters were reported to be homologous. Among these seven clusters, cluster12 (GME1619_g ~ GME1630_g), cluster28 (GME6528_g ~ GME6538_g), and cluster52 (GME2317_g ~ GME2323_g) had alignment results with 100% similarity rate. Furthermore, these three secondary metabolism gene clusters were functional characterized (Fig. 6B). Cluster12 (T1PKS) was predicted to have 100% similar to the known ochratoxin A (OTA) biosynthesis gene cluster in *Aspergillus steynii*, which is one of the most important mycotoxins with strong toxicity and worldwide distribution [30]. Cluster52 (terpene) was predicted to be homologous to the known ACR toxin biosynthetic gene in *Alternaria alternata*, which was required for the production of ACR toxin and the pathogenicity of *A. alternata* [63]. Cluster28 (NRPS-like) was capable to synthesis of choline which affects mycelial morphology by controlling branch initiation and is an essential metabolite for the growth of filamentous fungi [64]. In summary, these results suggest that SMs genes play an essential role in the development and infection of *P. elaeocarpicola*.

Fungal cytochrome P450 (CYP) monooxygenase superfamily plays a crucial role in a wide range of process, such as primary metabolism, secondary metabolism, detoxification, and virulence [65]. In this study, a total of 152 putative CYPs were predicted in *P. elaeocarpicola* (Table S4). Compared with typical pathogenic fungi *C. parasitica* (118 CYPs), *Verticillium dahliae* (69 CYPs), and *M. oryzae* (131 CYPs), *P. elaeocarpicola* encodes a great number of CYPs. CYP gene localization analysis showed that 25 CYP genes distributed in 19 secondary metabolism gene clusters (Table S3). To further predict the potential of CYPs in the pathogenic process of *P. elaeocarpicola*, 152 CYP genes were searched in the PHI-base. The results showed that a total of 102 CYP genes were similar to known fungal pathogenic CYP genes. Of these, six genes are similar to the characterized fungal CYP genes, such as VdCYP1, which is participate in the in phytotoxin (compound sulfacetamide) biosynthesis in *V. dahliae* [66] (Table S5).

### Determination of transporter proteins

Transporter proteins are a class of membrane proteins in which the ATP-binding cassette (ABC) and major facilitator superfamily (MFS) of transporters are common in filamentous fungi. Transporter proteins provide protection for filamentous fungi by resisting toxic compounds in the environment and transporting nutrients. In pathogenic fungi, membrane proteins can also mediate the secretion of host-specific toxins, against plant defensive compounds, and are important determinants in the pathogenic process [67]. Based on Transporter Collection Database [14], we identified 537 transporter genes in *P. elaeocarpicola* genome (Table S6).

MFS transporters are involved in the transport of a variety of substances such as organic and inorganic anions, monosaccharides and oligosaccharides, inositol, drugs, amino acids, organophosphorus esters and Krebs cycle metabolites. *P. elaeocarpicola* genome has the highest number of MFS transporters, and more than 60 MFS transporters were predicted, including Drug: H^+^ Antiporter-1 (DHA1), DHA2 and Sugar Porter (SP) family transporters (Table S7). Among them, SP family transporters are overrepresented in *P. elaeocarpicola* genome, with 23 genes, accounting for 37% of the MFS superfamily. This result suggested that *P. elaeocarpicola* might absorb more nutrients produced by host plants. Besides, the ability of DHA transporters to transport specific chemicals across the plasma membrane and to participate in the development of multidrug resistance suggest that *P. elaeocarpicola* can export more metabolites with high efficiency [68].

### Transcription factors (TFs) in the analyzed genomes

Transcription factors (TFs) are sequence-specific DNA-binding proteins required for the regulation of gene expression and can regulate a variety of biological processes, such as maintaining cellular homeostasis in response to environmental changes, regulating growth and development, and interacting with other organisms [69, 70]. In pathogenic fungi, TFs act as upstream components that regulate various pathways associated with fungal virulence, including hyphal growth, sporulation, secondary metabolite biosynthesis, infection-related morphogenesis, melanization and so on [70]. In current study, *P. elaeocarpicola* encodes 417 putative TFs, including 191 Zn2Cys6-type TFs, 82 C2H2-type TFs, 21 bZIP-type TFs, five GATA-type TFs, five APSES (bHLH) -type TFs and so on (Table S8). These TFs families play important roles in fungal development and pathogenicity [70].

To further investigate the role of TFs on the pathogenicity of *P. elaeocarpicola*, a total of 304 fungal development and pathogenicity related TFs were searched in the PHI-base. Eventually, 284 TFs were found to have homologs in PHI-base of which 66 TFs showed high similarity to known pathogenic genes (Table S9). For example, two putative Zn2Cys6-type TFs, *GME10067_g* and *GME6095_g*, were significantly similar to *GPF1* (PHI:3307) and *CCA1* (PHI:3314) of *M. oryzae*, respectively. Knockout *GPF1* and *CCA1* were experimentally proven resulted in loss of pathogenicity in *M. oryzae* [71]. In addition, two putative bZIP-type TFs, *GME8542_g* and *GME9227_g*, were similar to virulence associated genes *TOXE* (PHI:233) in *Bipolaris zeicola* and MoAP1 (PHI:2142) in *M. oryzae*, respectively [72, 73]. The other virulence associated TFs including the putative APSES-type TFs *GME4946_g* (Mstu1, PHI:2065, *M. oryzae*, loss of pathogenicity), the putative GATA-type TFs *GME766_g* (*clnr1*, PHI:287, *Colletotrichum lindemuthianum*, loss of pathogenicity) and three putative C2H2-type TFs *GME1376_g* (Con7p, PHI:2039, *M. oryzae*, loss of pathogenicity), *GME1536_g* (Vph1, PHI:7330, *V. dahliae*, loss of pathogenicity) and *GME7593_g* (Rbf1, PHI:2237, *Ustilago maydis*, loss of pathogenicity) [74–78]. These results suggest that the TFs of *P. elaeocarpicola* have great potential to participate in regulation of the fungal virulence.

## Discussion

*P. elaeocarpicola* is one of the most destructive pathogenic fungi of *Elaeocarpus* spp. in Guangdong Province, China. Until now, no effective treatment or detailed pathogenic mechanisms has been established for this disease. We firstly sequenced the genome of *P. elaeocarpicola*. The high-quality genome sequence of *P. elaeocarpicola* is 44.49 Mb, which is similar to that of *C. parasitica* (43.9 Mb) in the same family. The genome analysis provided us with a repertoire of genes related to *P. elaeocarpicola* infection and colonization of the host, including genes involved in the degradation of the plant cell wall, biosynthesis of secondary metabolites, synthesis of secreted proteins and translocation factors. This study lay the foundation for future research on *P. elaeocarpicola* and provide important data for the design of efficient control strategies.

The CAZymes secreted by *P. elaeocarpicola* fraction include many enzymes involved in the degradation of key plant cell wall components. In addition to GH16, *P. elaeocarpicola* also showed a marked expansion in GH5, GH43, and GH28 families compared with other tested fungi. Currently, one of the largest cellulase families is GH5, with at least 56 subfamilies [79]. Because GH5 family was the first cellulase family to be described also known as “cellulase family A”. GH5 family is also the basis for hemicellulose degradation. *Heterobasidion irregulare* possesses two GH5 (b-mannanases), which play an important role in the degradation of softwood hemicellulose [80, 81]. GH43 family is also a large family in GH families, which is divided into 37 subfamilies. The xylanolytic enzyme of GH43 is involved in the breakdown of hemicellulose [82]. These results demonstrate the powerful ability of *P. elaeocarpicola* to degrade cellulose and hemicellulose in host plant cell walls. GH28 family is an important extracellular enzyme in pathogenic fungi that hydrolyzes the glycosidic bonds in pectin, the main structural component of plant cell wall [83]. Many GH28 family members have been found to play critical roles in virulence in a variety of pathogenic fungi: VdEPG1 in *V. dahliae* can modulate the biosynthesis of jasmonic acid (JA) by regulating *GhOPR9*, and act as a virulence factor to regulate the host immune response. GH28 family also play an important role during the infection of *Xanthomonas campestris* pv. *campestris*, *Alternaria citri* and *B. cinerea* [84, 85]. In addition, as shown in Table S1, the number of genes for GH28 was generally higher in necrotrophic pathogens than in fungi of other lifestyles. This result may be caused by differences in fungal lifestyles. Necrotrophic pathogens vandalize cell wall components and degrade plant tissues to obtain carbon and energy. In contrast, biotrophic fungi will colonize intact plant cells while avoiding immune responses [86]. GH28, as the extracellular enzymes, trigger a series of plant immunological responses and stimulate host plants to produce hypersensitive immune responses or localized cell death mediated by polygalacturonase-inhibiting proteins (PGIPs) [83]. As a result, necrotrophic pathogens would appear to have a higher GH28 abundance than biotrophic fungi. In addition, previous studies have found that polygalacturonases of GH28 family contribute to the enhancement of virulence in *C. parasitica* [87]. Based on this result, it is highly likely that polygalacturonases belonging to the GH28 family in *P. elaeocarpicola* also contributes to virulence.

Previous studies on the interaction between pathogenic fungi of the Cryptomeriaceae family and host have found that the pathogenic fungi of this family have the ability to colonize through bark wounds [3]. In current study, CAZymes annotation results of *P. elaeocarpicola* are consistent with this feature of the family, and the significantly expand *P. elaeocarpicola* of the GH5, GH43, and GH28 family has great potential for colonization on host bark. Furthermore, based on the abundance of cellulose and hemicellulose degrading enzymes, *P. elaeocarpicola* has a higher capacity for plant cell wall degradation than *C. parasitica*, which is consistent with the fact that *P. elaeocarpicola* and *C. parasitica*, have different disease development time. Most diseased trees infected with *P. elaeocarpicola* died within five months, and smaller branches or twigs infected with *C. parasitica*, died within several months [3, 5].

Secreted proteases and membrane transporters play an important role in degradation of host structural proteins and nutrient absorption during plant infection, respectively. Our results revealed that *P. elaeocarpicola* genome contains numerous and functionally rich secreted proteins and effector proteins. Among the 171 effector proteins, we annotated four GH7 family proteins and 12 GH28 family proteins, of which GH28 family proteins has the highest number of proteins. Although the function of GH7 family proteins in plant pathogenic fungi is scarcely reported, the absence of PsGH7a has an indispensable influence on *Phytophthora sojae* virulence, which can lead to a reduction in the size of the lesion [88]. In addition, the *P. elaeocarpicola* secretome contains homologs of identified virulence secreted proteins of other plant pathogens as well as other pathogenesis-associated GH family members, including Gel1 and *Mo*PMO9A in *M. oryzae*, *SsCut1* in *S. sclerotiorum*, and so on (Table S2). Gel1 deficient mutant of *M. oryzae* could not produce conidia, and loss pathogenicity [52]. *Mo*PMO9A, which is significantly expressed in the early stages of infection, promotes the binding of CBM to the fungal cell wall chitin, which in turn weakens the plant cell wall and facilitates pathogen invasion [51]. Besides, *GME9214_g* in *P. elaeocarpicola* has a high homology with *SsCut1* in *S. sclerotiorum*. *SsCut1* gene enhances the pathogenicity of *S. sclerotiorum* through the enhancement of cutinase activity [55]. These predicted virulence-related proteins have a high likelihood of being involved in the pathogenic process of *P. elaeocarpicola*, and therefore can serve as target proteins to provide basic research materials for future studies of pathogenesis. The specific secreted protein of *P. elaeocarpicola*, GME8245_g, is homologous to VmEP1 of *V. mali* in PHI-base. VmEP1 is able to manipulate plant immune mechanisms by inhibiting BAX-induced programmed cell death and targeting host pathogenesis-related protein 10 (PR10) [89]. Based on this result, GME8245_g can be used as a target gene for interactions between pathogenic fungi and hosts for intensive study. Membrane transporters of pathogenic fungi could mitigate the damaging effects of defense compounds produced by host plants and transport nutrients. The *P. elaeocarpicola* genome contains more MFS superfamilies compared with ABC transporter families. Therefore, we speculate that the selective amplification of MFS superfamily and the loss of ABC transporters family in *P. elaeocarpicola* suggests that *P. elaeocarpicola* may possess multidrug resistance genes to combat specific antifungal compounds in ecological niche.

Secondary metabolites (SMs, also known as natural products), are produced by specific fungal taxa during secondary metabolism. SMs are important in fungal development and response to stressors (both abiotic and biotic) [29]. In phytopathogenic fungi, SMs can also enhance the infectiveness, and further improve the infection processes, such as Aflatoxin B, T-toxin, cercosporin toxin, fusaoctaxin A, and so on [32, 33, 90–92]. Our annotation shows that there are a large number of secondary metabolism gene clusters in *P. elaeocarpicola*, containing a total of 86 secondary metabolism backbone genes. Besides, cluster12 and cluster52 of *P. elaeocarpicola* were predicted to have 100% homology to the known toxin synthesis gene clusters ochratoxin A (OTA) and ACRTS2, respectively. OTA, mainly produced by *Aspergillus* and *Penicilum* species, is one of the most important mycotoxins that contaminates agricultural including cereal products, grapes and grapes products, cocoa, nuts, coffee, beverages, dried fruits, and cured meats [93]. OTA can cause many adverse effects on the health of humans and livestock, such as strong nephrotoxicity, hepatotoxicity, teratogenicity, immunosuppression and so on [93–95]. As for ACR-toxin, it is a host-selective toxins (HSTs) produced by *Alternaria* species during infection, which causes leaf necrosis of rough lemon and is a decisive factor in pathogenicity [96]. Previous reports showed that *C. parasitica*, which is closely related to *P. elaeocarpicola*, could produces a range of compounds associated with virulence, including oxalic acid, tannases, laccases, cryparin and diaporthin [87]. In summary, functionally rich SMs are likely to play important roles in *P. elaeocarpicola* growth and development, pathogenicity, stress response and other cellular processes.

## Conclusions

Taken together, the present study is the first to sequence and report the genome of *P. elaeocarpicola*, the pathogenic fungus of *Elaeocarpus* spp. stem blight. Intensive analysis of this genome suggests that *P. elaeocarpicola* may employ a variety of virulence strategies, including the secretion of CAZymes, secreted proteases, SMs, and membrane transporters, during the process of colonization and infection. Both CAZymes and SMs genes were significantly expanded in *P. elaeocarpicola* compared to the majority of typical reference pathogenic fungi. The *P. elaeocarpicola* genome provides valuable basic research materials for the molecular pathogenesis study and control strategies of blight pathogens.

## Materials and methods

### Biological material, genomic DNA extraction

*Pseudocryphonectria elaeocarpicola* was originally isolated from diseased *Elaeocarpus apiculatus* barks (collection of *Elaeocarpus apiculatus* barks complied with local guidelines). The strain were deposited in China Forestry Culture Collection Center (CFCC, http://cfcc.caf.ac.cn/), and the specimens in the herbarium of the Chinese Academy of Forestry (CAF, http://museum.caf.ac.cn/). Genomic DNA was extracted by the protocol of Cetyltrimethylammonium Bromide (CTAB). The integrity, purity, and concentration of genomic DNA were measured using Qubit fluorometer, and agarose gel electrophoresis, respectively.

### Genome sequencing, assembly and component prediction

Genomic DNA was extracted and randomly interrupted, DNA fragments of the desired length were recovered by electrophoresis, and splices were added for cluster preparation, and finally sequenced on the machine. Subsequently, the DNBSEQ platform sequentially removes 40% of the reads with consecutive bases ≤ 20 in the original sequencing data, reads with a certain proportion of Ns (10% as default), adapter contamination, and duplication contamination, and finally obtains Clean Reads. The raw data from PacBio sequencing are Polymerase Reads, which are filtered out to obtain usable Subreads and saved in bam format, such as sequencing splices and low-quality data. Consistent sequence analysis is performed on each ZMW to obtain more accurate data, i.e., circular consensus sequencing data (CCS, also known as insertion reads), which will be used directly for assembly, alignment, species classification, etc. Subreads less than 1000 bp in length are removed [97–99]. Sequencing data were assembled using a variety of software and can be divided into four sections: a. Subreads correct (Canu v1.5, FalconConsensus, SMRT Analysis v2.3.0) ;b. Corrected Reads Assembly (Canu v1.5, Falcon v0.3.0); c. Correct single base (GATK v1.6-13); d. Link Contig to Scaffold andfill gap (SSPACE_Basic v2.0), resulting in highly confident assembled sequences [98, 100–102].

The genetic components of *P. elaeocarpicola* were predicted using the software Augustus v3.2.1 and GeneMarkes v4.21 [103, 104].The rRNA was predicted by RNAmmer v1.2 ; the area of tRNA and its secondary structure were predicted by tRNAscan v1.3.1; and the sRNAs was obtained by Infernal software, which was compared with the Rfam database v9.1 [105–107].Transposon sequences were predicted by RepeatMasker v4-0-6, RepeatProteinMasker, and buildXDFDatabase (a denovo method). Tandem Repeat sequences (TR) were predicted by Tandem Repeat Finder v4.04 [108].

### Annotation of specific gene categories

In the current study, GO (2019-07-01) and KEGG (101) were used to analyze the GO category and pathway [109–111]. To identify secreted proteins of fungal, protein sequence of *P. elaeocarpicola* were subjected to the SignalP 4.1 and TMHMM 2.0. Based on these prediction results, EffectorP 3.0 was further used to predict effector proteins [112]. Transport proteins of fungal species in this study were predicted using TransportDB [14]. The transcription factors of *P. elaeocarpicola* were annotated based on IPRSCAN annotation combined with the collection of transcription factor-related IPR Numbers from Fungal Transcription Factor Database (FTFD). Align the putative CYP genes and transcription factors of *P. elaeocarpicola*, with the pathogen-host interaction database (PHI-base, http://www.phi-base.org/) to screen the potential fungal development and pathogenic related genes [113]. Gene density, GCskew, GCratio analysis, synteny analysis, and circle plot visualization were completed by TBtools [114]. The amino acid sequences of genes were compared with the carbohydrate-active enzyme database to identify genes encoding putative carbohydrate-active enzymes [46].

### Gene family analysis

The reference species genomic data used in this study were downloaded from the NCBI website (https://www.ncbi.nlm.nih.gov/genome/) (Table S10). Orthofinder v2.5.5 was used to identify the single-copy orthogroup within *P. elaeocarpicola* and all reference fungi [115]. Multiple sequence alignments of each single-copy orthogroup were performed using MAFFT v7.520 [116]. The maximum-likelihood (ML) tree was inferred with FastTree v2.1.11 [117]. The obtained phylogenomic tree was visualized with FigTree v1.4.4.

### Secondary metabolism genes and clusters analysis

The secondary metabolism gene clusters of *P. elaeocarpicola* were predicted using the antiSMASH fungal v7.0 [10]. The CYPs genes were predicted using Cytochrome P450 Engineering Database [12]. The domain structures of cluster12, cluster28, and cluster52 were annotated using the InterProScan tool (https://www.ebi.ac.uk/interpro/).

### Accession codes

The complete genome of *Pseudocryphonectria elaeocarpicola* is available at CNCB (China National Center for Bioinformation https://www.cncb.ac.cn) with accession numbers: GWHESHB00000000.

### Supplementary Information

Additional file 1: Figure S1. Gene Ontology (GO) terms of secretory proteins in *P. elaeocarpicola* genome.

Additional file 2: Table S1. Number of carbohydrate-active enzyme modules of *P. elaeocarpicola* and 12 other fungi according to the CAZy database.

Additional file 3: Table S2. Effector proteins annotated in the NR database.

Additional file 4: Table S3. Inventory of secondary metabolism gene clusters in *P. elaeocarpicola*.

Additional file 5: Table S4. Inventory of Cytochrome P450 related enzyme (CYPs) in *P. elaeocarpicola*.

Additional file 6: Table S5. Putative CYP genes showing homology to known fungal CYP genes.

Additional file 7: Table S6. Distribution of genes coding for membrane transporter families in *P. elaeocarpicola*.

Additional file 8: Table S7. Distribution of genes coding for family of the Major Facilitator Superfamily (MFS) in *P.elaeocarpicola*.

Additional file 9: Table S8. Inventory of Transcription Factors (TFs) in *P. elaeocarpicola*.

Additional file 10: Table S9. Predicted results of pathogenesis-related transcription factors (TFs) in the PHI-base.

Additional file 11: Table S10. Reference fungi information.

## Data Availability

The complete genome of Pseudocryphonectria elaeocarpicola is available at CNCB (China National Center for Bioinformation https://www.cncb.ac.cn ) with accession numbers: GWHESHB00000000.

## Abbreviations

CAZymes: Carbohydrate-active enzymes
SM: Secondary metabolite
NRPS: Non-ribosomal peptide synthetase genes
PKS: Polyketide synthase
CYPs: Cytochrome P450 related enzyme
GO: Gene ontology
KEGG: Kyoto encyclopedia of genes and genomes
PHI-base: Pathogen-host interactions database
MFS: Major facilitator superfamily
ABC: ATP-binding cassette
GHs: Glycoside hydrolases
GTs: Glycosyltransferases
PLs: Polysaccharide lyases
CEs: Carbohydrate esterases
CBMs: Carbohydrate-binding modules
AAs: Auxiliary activities
NR: Non-Redundant Protein Database
PG: Polygalacturonases
RG: Rhamnogalacturonases
XG: Xylogalacturonases
OTA: Ochratoxin A
DHA: Drug:H^+^ Antiporter-1
SP: Sugar Porter
TFs: Transcription factors
HSTs: Host-selective toxins
PGIPs: Polygalacturonase-inhibiting proteins
CTAB: Cetyltrimethylammonium Bromide
