# Supplementary figures and images for: Genome sequencing of Elaeocarpus spp. stem blight pathogen Pseudocryphonectria elaeocarpicola reveals potential adaptations to colonize woody bark
Source: BMC Genomics. 2024 Jul 24;25:714. doi: 10.1186/s12864-024-10615-5 (PMC11267912; doi:10.1186/s12864-024-10615-5)

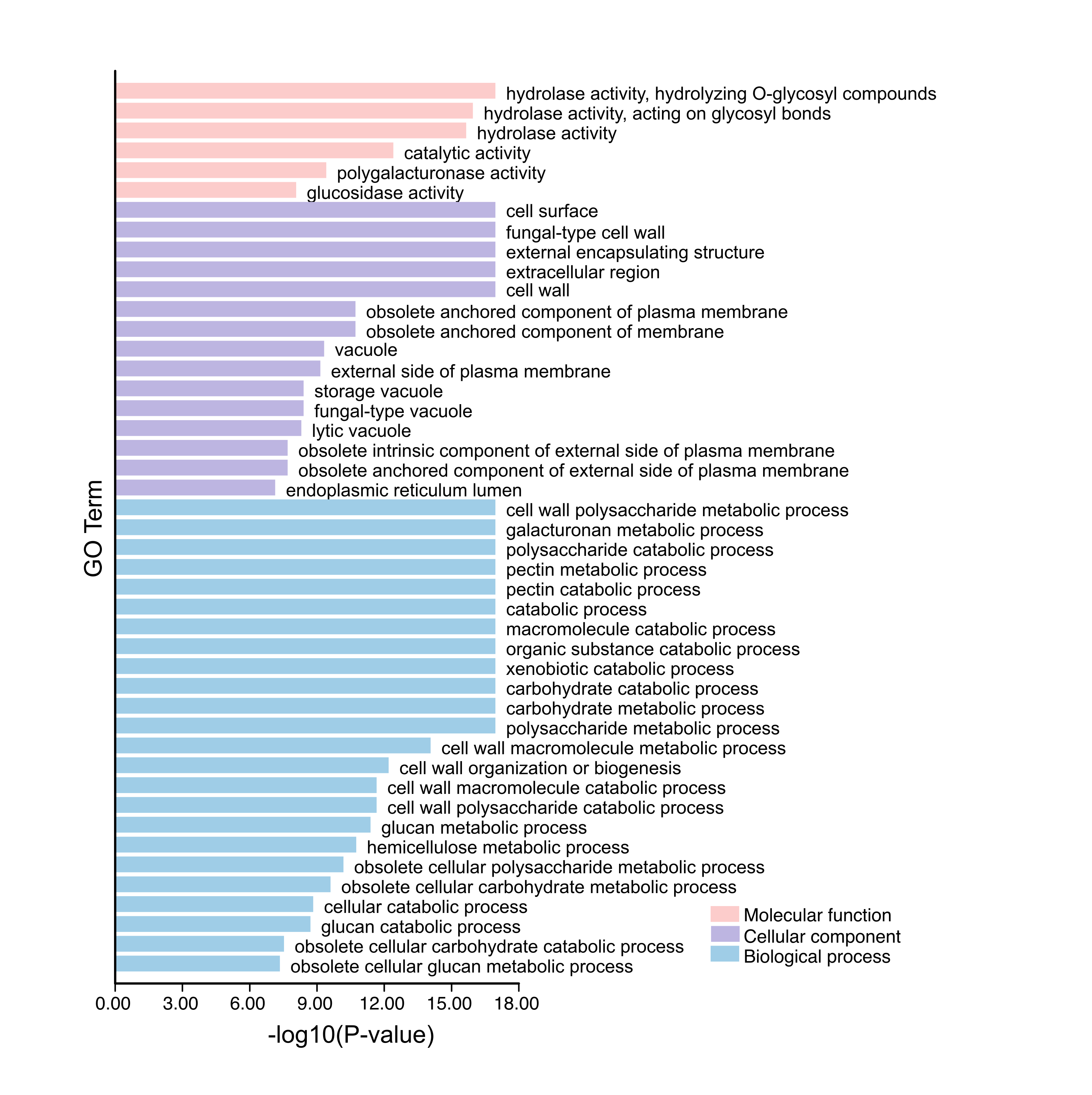

Supplement: Supplementary file 1 — Additional file 1: Figure S1. Gene Ontology (GO) terms of secretory proteins in P. elaeocarpicola genome. [file 12864_2024_10615_MOESM1_ESM.tif]
